# A Rapid SARS-CoV-2 RT-PCR Assay for Low Resource Settings

**DOI:** 10.3390/diagnostics10100739

**Published:** 2020-09-24

**Authors:** Arunkumar Arumugam, Matthew L. Faron, Peter Yu, Cole Markham, Michelle Wu, Season Wong

**Affiliations:** 1 AI Biosciences, Inc., College Station, TX 77845, USA; arunkumar.arumugam@aibiosciences.com (A.A.); mydearpeter@gmail.com (P.Y.); markhamc@gmail.com (C.M.); michellewu124@gmail.com (M.W.); 2 The Medical College of Wisconsin, Milwaukee, WI 53226, USA; mfaron@mcw.edu

**Keywords:** SARS-CoV-2 virus, RNA, rapid RT-PCR assay, low resource setting, thermal cycler

## Abstract

Quantitative reverse transcription polymerase chain reaction (RT-qPCR) assay is the gold standard recommended to test for acute SARS-CoV-2 infection. However, it generally requires expensive equipment such as RNA isolation instruments and real-time PCR thermal cyclers. As a pandemic, COVID-19 has spread indiscriminately, and many low resource settings and developing countries do not have the means for fast and accurate COVID-19 detection to control the outbreak. Additionally, long assay times, in part caused by slow sample preparation steps, have created a large backlog when testing patient samples suspected of COVID-19. With many PCR-based molecular assays including an extraction step, this can take a significant amount of time and labor, especially if the extraction is performed manually. Using COVID-19 clinical specimens, we have collected evidence that the RT-qPCR assay can feasibly be performed directly on patient sample material in virus transport medium (VTM) without an RNA extraction step, while still producing sensitive test results. If RNA extraction steps can be omitted without significantly affecting clinical sensitivity, the turn-around time of COVID-19 tests, and the backlog we currently experience can be reduced drastically. Furthermore, our data suggest that rapid RT-PCR can be implemented for sensitive and specific molecular diagnosis of COVID-19 in locations where sophisticated laboratory instruments are not available. Our USD 300 set up achieved rapid RT-PCR using thin-walled PCR tubes and a water bath setup using sous vide immersion heaters, a Raspberry Pi computer, and a single servo motor that can process up to 96 samples at a time. Using COVID-19 positive clinical specimens, we demonstrated that RT-PCR assays can be performed in as little as 12 min using untreated samples, heat-inactivated samples, or extracted RNA templates with our low-cost water bath setup. These findings can help rapid COVID-19 testing to become more accessible and attainable across the globe.

## 1. Introduction

Quantitative reverse transcription polymerase chain reaction (RT-qPCR) assay is the gold standard recommended to test for acute SARS-CoV-2 infection [1,2,3,4,5,6,7,8]. It has been used by the Centers for Disease Control and Prevention (CDC) and several other companies in their Emergency Use Authorization (EUA) assays [7,9]. Despite its established performance in sensitivity and specificity, RT-qPCR requires expensive equipment such as RNA isolation instruments and real-time PCR thermal cyclers, which are not available in many resource limiting settings. Moreover, even well-equipped labs also experience bottlenecks that could limit throughput.

As a pandemic, COVID-19 has quickly spread indiscriminately. Many underdeveloped and developing counties do not have the means for fast and accurate COVID-19 detection to control this outbreak. Our approach updates the archaic method of hand-transferring reaction tubes through a series of water baths. We constructed two water baths from plastic containers that were heated by sous vide immersion heaters. These heaters are easy to use, have precise temperature control and circulate the water for even heating. One heater is used for the denaturation step and the second for the reverse transcription and annealing/extension steps. The PCR tubes are then shuttled between water baths using a servo-motor operated arm controlled by a programmable microcontroller device [10,11,12]. The unit presented here can process up to 96 samples in one run and only costs approximately USD 300 to build.

To further improve the speed of a diagnostic assay, we and others tested using untreated or heat-inactivated samples added directly to one-step RT-PCR master mixes without an RNA extraction step [6,13,14,15,16,17,18,19]. With many PCR-based molecular assays, an extraction step is routinely used as part of the protocol. This step can take up a significant amount of time and labor, especially if the extraction is performed manually. Long assay time, partly caused by slow sample preparation steps, has created a large backlog when testing patient samples suspected of COVID-19. Our findings in this paper with COVID-19 clinical samples suggest that it is possible to eliminate the RNA extraction step in COVID-19 testing without a significant drop in assay sensitivity for samples from symptomatic patients. Eliminating this extraction step can potentially improve the throughput of testing patients with acute infection symptoms whose viral load tends to be very high (under a threshold cycle of 30 when tested by RT-qPCR). Numerous studies have looked at the SARS-CoV-2 viral load in specimens of infected patients, and high viral loads were detected soon after symptom onset [20,21,22,23,24,25,26,27,28,29,30,31,32,33]. When viral load is high, the effect of inhibitors can be masked, and RT-PCR can still produce a positive result (e.g., Ct <40), even if full RNA extraction is not used.

## 2. Materials and Methods

### 2.1. Materials and Reagents

The AccuPlex SARS-CoV-2 reference material kit (Cat. No. 0505-0126) was purchased from SeraCare (Milford, MA, USA). Primer and probe sets for the SARS-CoV-2 RT-qPCR assay (Cat. No. 10006606) were purchased from Integrated DNA Technologies (Coralville, IA, USA). The clinical specimens of Influenza A (DLS16-85584), Influenza B (DLS15-33890), and RSV (KH19-00715) were obtained from Discovery Life Sciences Inc. The TaqPath 1-step multiplex master mix (Cat. No. A28525) was purchased from Thermo Fisher Scientific (Waltham, MA, USA). The viral transport media (Cat. No. R99) used for the dilution and spiking experiments was purchased from Hardy diagnostics (Santa Maria, CA, USA).

### 2.2. Preparation of Low Viral Load Clinical Specimen Mimics

AccuPlex SARS-CoV-2 reference material from SeraCare containing non-replicative viral particles (5000 viral particles per mL) was mixed with equal volumes of virus transport medium (VTM), to get a final concentration of 2500 viral particles per mL. Each microliter contained ~2.5 genomic material equivalents of SARS-CoV-2.

### 2.3. Preparation of High Viral Load Contrived Clinical Specimen to Test the Effect of VTM on RT-qPCR

SARS-CoV-2 positive control plasmid (CDC recommended) was obtained from Integrated DNA Technologies (200,000 copies/µL). This was diluted to 10,000 copies/µL in TE buffer and was used as a stock solution. 10 µL of this stock solution was added to 990 µL of VTM to get a final concentration of 100 copies/µL (VTM working solution). A control was prepared by diluting 10 µL of the stock solution in 990 µL of TE buffer (TE buffer working solution at 100 copies/µL). Four microliters of the VTM or TE buffer working solutions were added into each 20-µL PCR reaction.

### 2.4. Automated Sample Preparation

Automated nucleic acid extraction was performed in a Promega Maxwell device using AS1520 cartridge (Promega Corporation, Madison, WI, USA). For each COVID-19 sample from MCW, 60 µL were added to each cartridge and placed in the extraction device for automated processing. The entire protocol took about 40 min and extracted nucleic acids were eluted in 60 µL of the elution buffer provided in the kit.

### 2.5. Rapid Extraction

We have used a rapid sample preparation protocol to reduce the sample-to-test time. A total of 60 µL of each COVID-19 sample from MCW was added to 400 µL of lysis buffer and 15 µL of magnetic particle solution (NucliSENS Magnetic Particle Extraction Kit, bioMerieux, Durham, NC, USA), and lysed for 1 min. The magnetic particles were collected and washed in the kit’s wash buffers 1 and 2 for 30 s each. Next, the magnetic particles were air-dried for 1 min and then eluted in 60 µL of elution buffer at 75 °C for 1 min. This 4-min automated protocol was performed on an in-house built sample preparation device [34,35].

### 2.6. Using Sous Vide Immersion Heaters to Create a Portable and Low-Cost Alternative to Circulating Water Baths Used in Laboratories

We have previously devised a low-cost alternative to a thermal cycler using the thermos thermal cycler (TTC), a PCR method using thermos cans as insulated water baths to create a semi-automated low-cost alternative to conventional thermal cyclers to be used in low resource settings and small laboratories [11,12]. Water temperature was maintained at denaturation and annealing/extension temperatures for the duration of each PCR run without active heating and cooling control.

**Figure 1:**
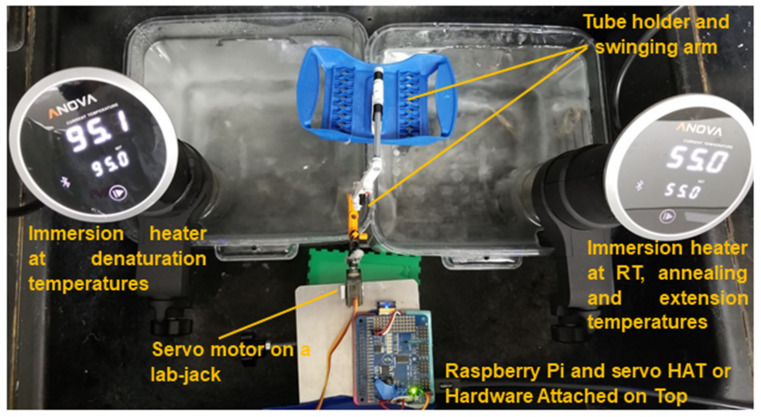
Water bath setup for SARS-CoV-2 detection using reverse transcription polymerase chain reaction (RT-PCR). Sous vide immersion heaters provided sufficient and consistent temperatures for RT, denaturation, and annealing/extension steps. A Raspberry Pi controlled a servo motor that moved the PCR tubes between the baths with a cell phone app via Wi-Fi connection. Large containers enable large number of samples to be tested.

For the current work, to achieve a steady circulating water system with consistent temperatures for denaturation and annealing/extension steps, we used two sous vide immersion heaters purchased at USD 99 each (Anova Culinary, 800 watt) to heat the water in two 6 quart clear food storage containers (Rubbermaid) to 55 °C and 95 °C, both of which are common PCR annealing/extension and denaturing temperatures (Figure 1). The temperature of the annealing/extension bath was first lowered to 53 °C during the reverse transcription step. We have previously determined that the water bath temperature remained steady at a set temperature (97 °C and 60 °C), with only 0.1 °C of variation (Figure S1). The sous vide immersion heater is a medium-sized device that can be securely clamped onto the edge of the food storage containers. Its steady heating and water circulation functionality results in a well-maintained, even temperature throughout the bath for long periods of time. We were able to measure the temperature throughout the heating duration using a temperature data logger (HH147U, Omega Engineering, Norwalk, CT, USA).

### 2.7. Automation Using a One-Servo Device

Automation of the thermal cycling reactions was achieved by programming a one-servo motor and devising a holder assembly with LEGO pieces to shuttle PCR tubes between the water baths (Figure 1). An arching movement was used to lift, transport, and lower the tubes between two baths in a single semi-circle motion. The tubes were contained in a hinged holder that would allow the tubes to remain upright throughout the movement phase. The mechanical portion was driven by a micro servo (MG90S Micro Servo Motor, Amazon) and was controlled by a microcontroller (Raspberry Pi Model A+) using a 16-Channel PWM/Servo Hardware Attached on Top (HAT) (Adafruit). Precise movement by the servo moved the arm back and forth between the two baths. The semi-circle movement of the arm, therefore, can move the PCR tubes in and out of the water. The entire arm assembly was mounted on a small lab-jack. A USB connector powered the servos and controller.

### 2.8. RT-qPCR Reaction Setup

TaqPath 1-step multiplex master mix (Thermo Fisher) was used for the RT-qPCR reaction with specific primers and probes labeled with fluorescein (FAM) dye. The final concentration of primers and probe sets for influenza A, B and RSV in the reaction is 250 nM in a 20 µL reaction [36,37]. For the detection of SARS-CoV-2 target genes, N1 and N2, the final concentration of primers was 500 nM and probes were 125 nM as per the CDC protocol (Table 1) [38,39]. The US CDC has already tested the primer sequences for cross-reactivity with SARS-CoV and 22 various respiratory tract pathogens, including human coronaviruses 229E, OC43, NL63, HKU1, SARS-CoV, and MERS-CoV [39]. They have found no cross-reactivity with the tested sequences. Others have also used these primers for their work [1,40,41].

Each PCR reaction was 20 µL with 3 µL samples/extracted template added. The typical run time for the Bio-Rad cycler to complete 50 cycles was 82 min when performed using a Bio-Rad CFX-96 real-time thermal cycler (Table 2). Specimens that produced Ct values of >40 in the real-time RT-PCR assay were considered negative samples.

The InfA, InfB, and RSV primers and probes used in this study were taken from previously published articles [36,37] and the SARS-CoV2 targeting primers and probes were taken from Centers for Disease Control Prevention, Coronavirus Disease 2019 (COVID-19) Real-time RT-PCR Panel Primers and Probes [38].

### 2.9. Rapid PCR with Water Baths

Rapid PCR was conducted using two water baths maintained at 95 °C and 55 °C (Table 3). Our goal was to gradually reduce the cycling time by a second or two without affecting the efficiency of PCR amplification. We did this by empirically reducing either the reverse transcription, denaturation, or annealing/extension time. We tested 5 to 12 s of denaturation time and 5 to 18 s of annealing/extension times. We judged the success of RT-PCR runs (with fluorescence signal higher than no template controls and negative controls) by looking at the intensity after the first 40 cycles. If the signal was still weak (indeterminate) after the first 40 cycles and adding additional cycles increased the fluorescence signal significantly, we can be certain that the efficiency of amplification has been compromised when the protocol was shortened too much.

In this work, we found that 6 s of denaturation and 9 s of annealing/elongation is sufficient for amplification of ~110 bp target (InfA) using a water bath. The amplicon size of SARS-CoV-2 N1 and N2 primers are 67 and 71 bp, so we speculated that 6 s and 9 s of PCR cycle would be sufficient to amplify these targets as well. We also tested the rapid RT-qPCR conditions with 5 copies of SARS-CoV-2 positive plasmid, and the RT-PCR run resulted in a positive signal. This means the current protocol did not compromise the RT-PCR efficiency in a way that would reduce the sensitivity.

In this work, reverse transcription was carried out for 90 s at 53 °C and initial denaturation was carried out for 30 s followed by 40 cycles of PCR amplification with 6 s of denaturation at 95 °C and 9 s of annealing/extension at 55 °C. The fluorescence intensity of the reaction mix was analyzed after amplification. The total time for PCR was approximately 12 min, including 2 min of RT and initial denaturation, and 10 min of PCR amplification for 40 cycles. It took 13.3 min for 45-cycle reactions (2 min of RT and initial denaturation and 11.3 min of PCR amplification). Protocols that deviated from these typical settings are noted in the results section. PCR tubes used in this study were Cepheid SmartCycler PCR reaction tubes (Cepheid, Sunnyvale, CA, USA) and thin-walled polypropylene PCR tubes (Cat. No. 16950, Sorenson Bioscience, Salt Lake City, UT, USA).

### 2.10. End-Point RT-PCR Fluorescence Analysis

After amplification by the water bath thermal cycler, PCR tubes were placed above a gel-viewing blue LED powered lightbox (Lonza Flashgel Dock or IO Rodeo Midi Blue LED Transilluminator) to confirm amplification through fluorescence intensity. Fluorescence across reactions was imaged using a smartphone camera (Note 8, Samsung Electronics), with an amber filter placed in front of the lens. We visually determined the RT-PCR result as positive or negative using the negative control sample tube as a reference. We also used ImageJ to analyze the fluorescence intensity of the PCR tubes after the amplification runs. The fluorescence value of each tube was calculated by subtracting the background signal outside of the tube from the sample area.

### 2.11. Ethics Statement

The intent of the work was for clinical method development as a response to the COVID-19 pandemic. In this work, we used anonymized remnant material from samples that had been collected for clinical diagnostics of SARS-CoV-2. Our SARS-CoV-2 positive clinical specimens were provided by the Medical College of Wisconsin (MCW). These samples included nasopharyngeal swabs in universal transport medium (UTM) and viral transport medium (VTM). These de-identified samples were collected under an IRB approved by a MCW IRB (#PRO00034003 approved on 3/26/2019). Other biospecimens were purchased from Discovery Life Science (San Luis Obispo, CA, USA). These de-identified remnant samples are not considered to be human subjects.

## 3. Results and Discussion

### 3.1. RT-qPCR Analysis of Ten COVID-19 Positive Clinical Samples Using Commercial Real-Time Thermal Cycler

To determine the relative viral load of the clinical samples, 60 µL of the specimen was extracted using an automated system (Promega Maxwell) and run on a commercial real-time thermal cycler. Three µL of the templates were used in each RT-qPCR reaction. The qPCR threshold cycle (Ct) values of these samples are listed in Table 4. It took the commercial cycler 1 h and 22 min to complete the reaction. Furthermore, to test the need for RNA extraction, 3 µL of each media was spiked into 17 µL of the master mix. As seen in Table 4, the 10 extracted templates produced Ct values of less than 40. Using unprocessed samples spiked directly into PCR master mix, the Ct values (also <40) show that the samples can be determined as positive. The results indicated that using unprocessed (direct spiking) transport media samples can produce qualitative PCR results matching those performed using extracted RNA templates. More importantly, even in the samples with low viral load (samples 1, 3, 8, and 9 with Ct > 30), the testing of unprocessed samples by a commercially available real-time thermal cycler produced positive results.

### 3.2. Rapid RT-PCR Detection of SARS-CoV-2 (N1 target) in Unprocessed Clinical Samples Using Water Baths

**Figure 2:**
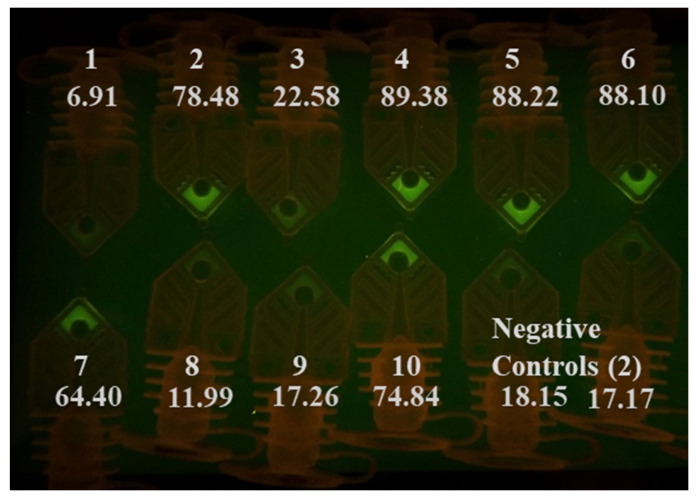
PCR tubes after a RT-PCR run using 3 µL of untreated transport media samples. Seven out of ten samples can be identified as positive COVID-19 patient samples by comparing results with negative controls. The RT-PCR run was completed in 12 min, and the RT-PCR protocol was (90 s/30 s/40 × (6 s/9 s)). The signal intensity measured by ImageJ is listed under the sample number.

Prior to COVID-19 emerged as a global pandemic, we have tested the feasibility of circumventing the sample preparation steps by adding a few microliters of the unprocessed sample (in VTM) directly into the RT-qPCR assay master mix targeting InfA, InfB, and RSV. We also performed RT-qPCR with unprocessed SARS-CoV-2 samples for this study. The addition of unprocessed InfA, InfB, and RSV samples in VTM was amplified in the thermal cycler, and the results are presented in Figure S2. Up to 6 µL of unprocessed InfA specimen in VTM did not inhibit the 20-µL PCR amplification process (Figure S3). The AccuPlex SARS-CoV-2 viral particles in its stock solution were mixed with an equal volume of VTM. 2, 4, 6, and 8 µL of this mixture were spiked into the master mix directly and amplified, along with extracted templates prepared using the same sample. Spiking of as low as 2 µL of unprocessed material in VTM was detected in the RT-qPCR reaction (Figure S4). We also tested the SARS-CoV-2 plasmid diluted in VTM and compared it with plasmid diluted in TE buffer. The RT-qPCR reaction was able to detect the plasmids in both VTM and TE buffer (Table S1). This indicates that the samples in VTM can be used directly in the RT-qPCR reaction and this approach can shorten the testing time without significantly impacting the test’s sensitivity, when applied to the samples of symptomatic patients whose viral load tend to be high [31]. We tested whether we could skip the extraction step and use unprocessed samples directly in a water-bath-based rapid RT-PCR test by spiking them into RT-PCR master mix targeting N1. We performed the initial reactions with a protocol of 90 s reverse transcription step, 30 s of reverse transcriptase inactivation, and a hot start for the PCR. This was followed by 40 cycles of 6 s of denaturation and 9 s of annealing/extension steps. The RT-PCR run was completed in 12 min (90 s/30 s/40 × (6 s/9 s)). Afterwards, photos of the PCR tubes were taken when illuminated on a blue LED gel box with an amber viewing filter (Figure 2). While we wanted to use visual inspection to call the test result positive or negative, we took the photos of the tubes and used ImageJ to measure the intensity of the samples after each RT-PCR run, to confirm the results and to determine if the visual inspection is reliable.

By comparing the tube intensity with the negative control with naked eyes, we were able to identify that 7 out of 10 of the samples as N1 positive (sample number 2, 3, 4, 5, 6, 7, and 10). Sample 3 was considered a weak positive. The signal intensity for samples 1, 8, and 9 was not significantly different from the negative controls shown in the photo. The signal intensity obtained from ImageJ analysis confirmed the conclusion judged by visual inspection. These three samples (1, 8, and 9) all produced Ct values over 30 when tested by commercial RT-qPCR (Table 4). We also speculate that the sensitivity of this run using unprocessed samples was low (70%), because we only spent 90 s on RNA reverse transcription. The inhibitors in the samples could have affected the cDNA production from the RT process, as well as the polymerase extension step. Though not presented here, we have also determined that ingredients in UTM inhibit our RT-PCR reactions more than VTM. We compared the RT-PCR inhibitory effect of unprocessed samples in VTM and UTM by directly adding them into the master mix. The results (not shown) showed that adding up to 7 µL of unprocessed samples in VTM to a reaction did not severely affect the Ct value. On the contrary, the addition of just 2 µL of the sample in UTM can completely inhibit the reaction in some cases. This can explain why samples 1, 8, and 9 (low viral load samples in UTM) did not test as positive. These results suggest that the assay sensitivity under these conditions (clinical specimens in UTM) is in the low 30 s Ct values. As a reference, we can detect five copies of SARS-CoV-2, positive control plasmid (CDC) per PCR reaction using primer targeting N2.

### 3.3. Rapid RT-PCR Detection of SARS-CoV-2 with N1, N2, and RNase P Reactions Using Water Baths

**Figure 3:**
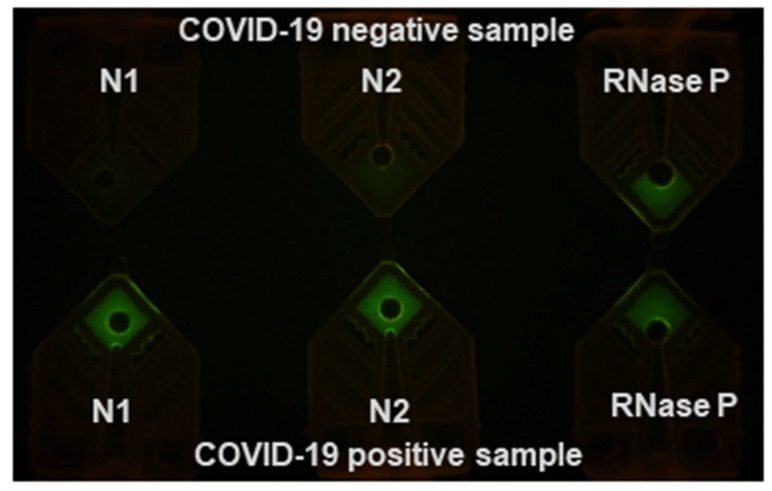
N1, N2, and RNase P in thin-walled PCR tubes after a RT-PCR run using 3 µL of extracted templates. Extracted template from AI007 sample, having medium viral load (Ct-29.1) was used. The total run time was 12 min (90 s/30 s/40 × (6 s/9 s)).

To test for SARS-CoV-2 using water bath based RT-PCR, we prepared three singleplex PCR reactions in Cepheid SmartCycler Tubes, each targeting N1, N2, and RNase P. Extracted templates from COVID-19 positive clinical specimens and contrived negative samples were tested. The 40-cycle RT-PCR runs were completed in 12 min using 3 µL of extracted templates. Figure 3 shows the PCR tubes on a blue LED gel box after 40 cycles as taken by a cell phone camera. In the COVID-19 positive sample (sample AI 007), all three tubes had an increased fluorescence signal after the reaction. In a COVID-19 negative sample, only the RNase P tube had increased fluorescence signal.

**Figure 4:**
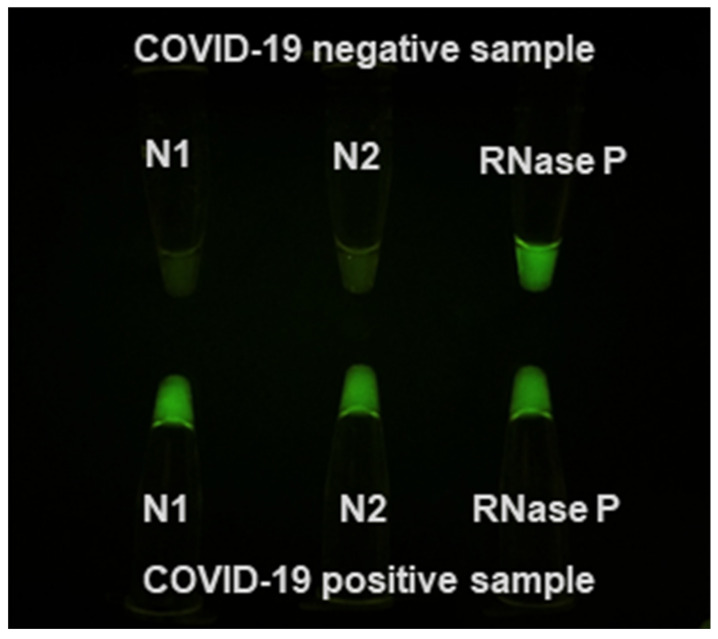
N1, N2, RNase P regular PCR tubes after RT-PCR reaction using 3 µL of extracted templates. Due to slower heat transfer, longer incubation time was needed. The total run time was 30 min (120 s/30 s/40 × (15 s/25 s)).

We repeated the work using thin-walled PCR tubes and 3 µL of extracted templates in 20-µL reactions. While the reaction took 30 min in total because of slower heat transfer when plastic with thicker wall compared to thin film was used (Sorenson Bioscience), the general trend matched those of the thin-film Cepheid PCR tubes (Figure 4).

### 3.4. Rapid RT-PCR Detection of SARS-CoV-2 in Heat-Inactivated Clinical Samples

**Figure 5:**
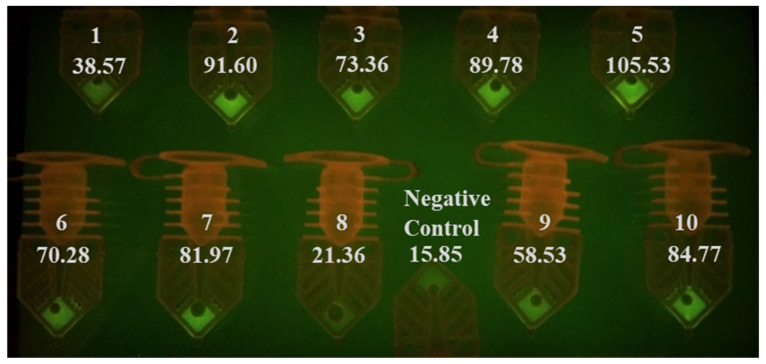
PCR tubes after a RT-PCR run using 3 µL of 10-min, 95 °C heat-inactivated samples. Nine out of ten samples can be identified as positive based on the fluorescence signal difference when compared to the negative control. The RT-PCR run was completed in 12 min (90 s/30 s/40 × (6 s/9 s)). The signal intensity measured by ImageJ is listed under the sample number.

To reduce inhibition and improve sensitivity, a 10-min, a 95 °C heat inactivation step was added to treat the samples. Three microliters of these samples were then added into RT-PCR master mix before performing the test (N1). The photo taken after 40 cycles shows a significant increase in fluorescence signal in nine out of 10 samples (Figure 5). Samples 1 and 9 were also determined as positive based on fluorescence increase. The signal intensity for sample 8, the sample with the highest threshold cycle value (Ct = 35.9) among the 10 samples when tested by a commercial real-time thermal cycler, was not different from the VTM-only negative control when viewed with the naked eye. The ImageJ data, however, showed that intensity from sample 8 was higher than that of the negative control. The improved sensitivity from using heat-inactivated samples for testing COVID-19 is similar to our results with flu sample testing (not shown), and from others reported recently [5,6,19,42].

### 3.5. RT-PCR Detection of SARS-CoV-2 with A 4-Minute Extraction Protocol

**Figure 6:**
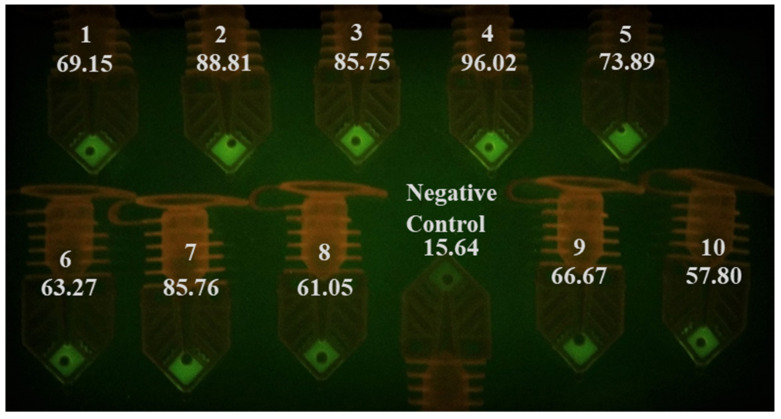
PCR Tubes after a RT-PCR run using 3 µL of extracted templates. Ten out of ten samples can be identified as positive based on the fluorescence signal difference when compared to the negative control. The RT-PCR run was completed in 12 min (90 s/30 s/40 × (6 s/9 s)). The signal intensity measured by ImageJ is listed under the sample number.

We next tested whether we could use a rapid RNA extraction step to produce RNA templates for rapid RT-PCR from raw samples without heat inactivation. We developed our own rapid magnetic particle-based extraction protocol using lysis buffer and two wash buffers before elution took place at 75 °C. Three microliters of the extracted templates were added to the RT-PCR master mix and amplified using the water bath method. The photo taken after 40 cycles shows that we can identify 10 out of 10 samples as N1 positive (Figure 6). ImageJ data confirmed the determination made by visual inspection with all 10 samples had a much high fluorescence signal over the negative control. The results suggest that a quick extraction step can further improve sensitivity further over heat-inactivated samples.

### 3.6. Limitation Statement

This study used a very limited number of clinical samples; therefore, it may be difficult to apply these results to other samples that might have a different amount of RNA and inhibitors in the samples. A larger number of samples will be needed to determine the sensitivity of using minimally processed samples vs. those processed with full RNA isolation and purification steps.

## 4. Conclusions

For rapid COVID-19 testing, using raw samples or minimal sample preparation steps may not significantly reduce the test sensitivity, as most patients tend to have a higher viral load when showing symptoms [20]. Therefore, we report that the use of untreated samples can be a viable option during the COVID-19 pandemic to speed up testing. Omitting extraction also allows personnel to divert that time towards other essential tasks. Furthermore, this approach might be suitable in pool testing COVID-19 specimens, allowing for the conservation of both material and human resources.

Using unprocessed samples with a 90 s reverse transcription step, we only were able to identify seven out of 10 samples as positive. Using raw samples after 10 min of heating at 95 °C, we were able to detect nine out of 10 samples using our approach. As expected, samples with a high viral load can easily be detected if the RT-PCR input uses raw or extracted templates. Samples with a low viral load or untreated COVID-19 positive samples are more likely to be missed if an extraction step is not used or when the PCR protocol is shortened when the presence of inhibitors affects the amplification efficiency. Using a 4 min RNA extraction protocol that we developed, we were able to get positive RT-PCR results with all ten COVID-19 positive clinical samples.

We also determined that using our USD 300 water bath set up for fluorescence-based end-point RT-PCR reactions can test for COVID-19 by running N1, N2, and RNase P RT-PCR reactions. This would allow locations with no access to real-time PCR thermal cyclers or even basic thermal cyclers to perform highly sensitive and specific gold-standard RT-PCR assays for COVID-19. Our post-RT-PCR photos of the PCR tubes show that the water bath-based RT-PCR allows for 40 cycle RT-PCR reactions to finish in as little as 12 min, with thin-walled Cepheid SmartCycler PCR tubes and 30 min with regular PCR tubes.

Companies such as Cepheid, Abbot, and Mesa Biotech have received Emergency Use Authorization (EUA) status on their rapid molecular COVID-19 tests. However, their users still need to buy instruments to perform the test. These cartridge-based assays are low throughput assays and need regular maintenance. Our format is simple to perform and does not require scheduled maintenance. It can be made readily available at physician’s offices, urgent care centers, pharmacies, or it can be used at home if needed. For countries without the infrastructure to perform rapid RT-PCR for SARS-CoV-2, our approach and setup is economical and highly feasible. Besides circumventing the limited availability of RNA extraction reagents and time-consuming process, we can perform 96 tests simultaneously using the rapid water bath-based RT-qPCR, as the water baths are large enough to process a 96-well PCR plate. The disadvantage of this approach is that a non-multiplexed reaction algorithm uses larger amount of master mix reagents. Our data suggest that rapid RT-PCR can be implemented for sensitive and specific molecular diagnosis of COVID-19 in situations where sophisticated laboratory instruments are not available.

## Figures and Tables

**Table 1:** Primer and probe sequences.

Primers	Sequence (5′–3′)	Annealing Temperature	Product Size
2019–nCoV_N1–F 2019–nCoV_N1–R 2019–nCoV_N1–Probe	5′–GACCCCAAAATCAGCGAAAT–3′ 5′–TCTGGTTACTGCCAGTTGAATCTG–3′ 5′–ACCCCGCATTACGTTTGGTGGACC–3′	55 °C	71 bp
2019–nCoV_N2–F 2019–nCoV_N2–R 2019–nCoV_N2–Probe	5′–TTACAAACATTGGCCGCAAA–3′ 5′–GCGCGACATTCCGAAGAA–3′ 5′–ACAATTTGCCCCCAGCGCTTCAG–3′	55 °C	67 bp
InfA Forward InfA Reverse InfA Probe	5′–GACCRATCCTGTCACCTCTGAC–3′ 5′–AGGGCATTYTGGACAAAKCGTCTA–3′ 5′–TGCAGTCCTCGCTCACTGGGCACG–3′	55 °C	106 bp
InfB Forward InfB Reverse InfB Probe	5′–TCCTCAACTCACTCTTCGAGCG–3′ 5′–CGGTGCTCTTGACCAAATTGG–3′ 5′–CCAATTCGAGCAGCTGAAACTGCGGTG–3′	55 °C	102 bp
RSV Forward RSV Reverse RSV Probe	5′–GGCAAATATGGAAACATACGTGAA–3′ 5′–TCTTTTTCTAGGACATTGTAYTGAACAG–3′ 5′–CTGTGTATGTGGAGCCTTCGTGAAGCT–3′	55 °C	84 bp
RNaseP Forward RNaseP Reverse RNaseP Probe	5′–AGATTTGGACCTGCGAGCG–3′ 5′–GAGCGGCTGTCTCCACAAGT–3′ 5′–TTCTGACCTGAAGGCTCTGCGCG–3′	55 °C	65 bp

**Table 2:** Quantitative reverse transcription polymerase chain reaction (RT-qPCR) setup in a commercial thermal cycler.

Process	Temperature	Time	Cycles
UNG incubation	25 °C	2 min	1
RT	50 °C	15 min	
Polymerase activation	95 °C	2 min	
PCR	95 °C	3 s	50
	55 °C	30 s	

**Table 3:** Rapid RT-PCR setup with water baths.

Process	Temperature	Time	Cycles
**RT**	53 °C	90 s	1
**Polymerase activation**	95 °C	30 s	
**PCR**	95 °C	6 s	40 or 45
	55 °C	9 s	

**Table 4:** Threshold cycle values of our COVID-19 positive samples tested by RT-qPCR.

Sample Number	Media	Threshold Cycle Values with Extracted Templates (*n* = 2)	Threshold Cycle Values with Unprocessed Samples (*n* = 1)
AI001	UTM	33.2	30.4
AI002	M6 VTM	23.0	23.4
AI003	UTM	30.1	31.9
AI004	UTM	24.9	26.3
AI005	M6 VTM	23.8	23.4
AI006	M6 VTM	27.7	24.6
AI007	M6 VTM	29.1	28.1
AI008	UTM	34.6	35.9
AI009	UTM	33.8	33.4
AI010	M4 VTM	25.9	24.9
Negative Control	VTM	N/A	N/A

## Supplementary Materials

The following are available online at https://www.mdpi.com/2075-4418/10/10/739/s1, Figure S1. Maintaining denaturation and annealing/extension temperatures for RT-PCR, Figure S2. Direct detection of influenza A, B and RSV using unprocessed samples in VTM, Figure S3. Using clinical samples without RNA extraction steps did not inhibit the reaction, Figure S4. Low concentrations of SARS-CoV-2 reference material in VTM are detected without an RNA extraction step, Table S1: RT-qPCR detection of SARS-CoV-2 plasmid in VTM.
