# Supplementary material for: A Rapid SARS-CoV-2 RT-PCR Assay for Low Resource Settings
Source: Diagnostics (Basel). 2020 Sep 24;10(10):739. doi: 10.3390/diagnostics10100739 (PMC7598596; doi:10.3390/diagnostics10100739)
Supplement: Supplementary file 1 [file diagnostics-10-00739-s001.pdf]

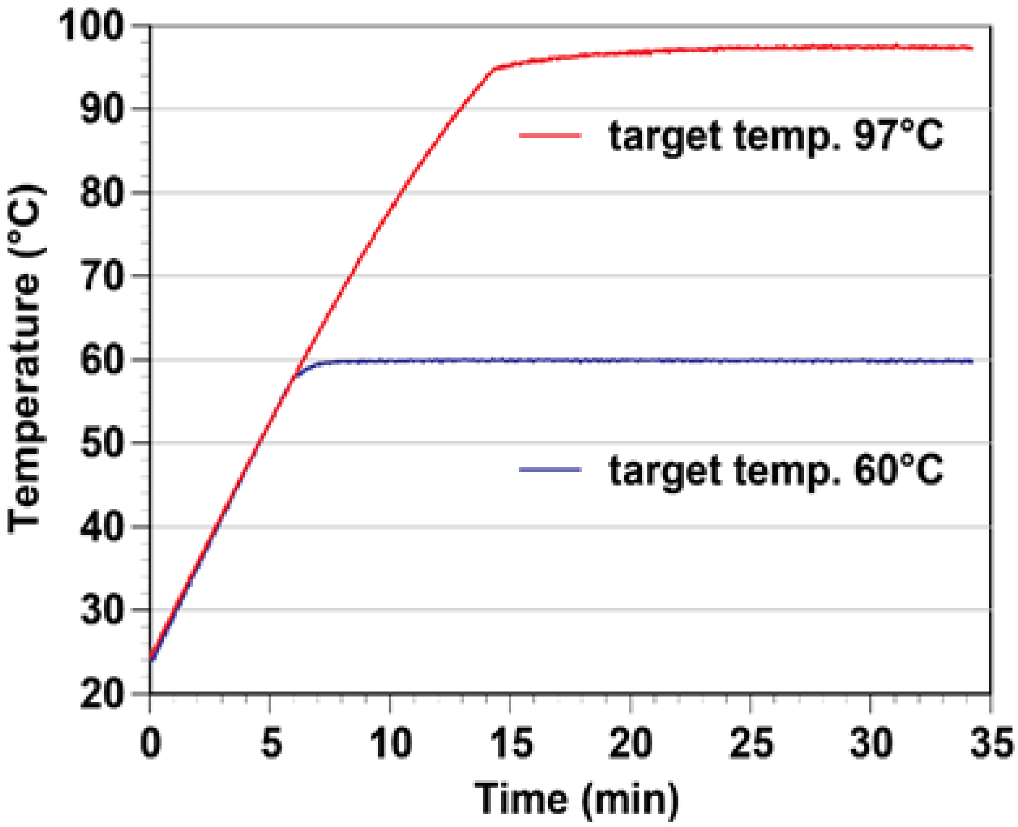

Figure S1. Maintaining denaturation and annealing/extension temperatures for RT-PCR. Sous vide immersion heaters were used to heat up water from room temperature to 60 °C and 97 °C. These immersion heaters can maintain water bath temperatures at denaturation and annealing/extension temperatures steady enough for the duration of thermal cycling reactions.

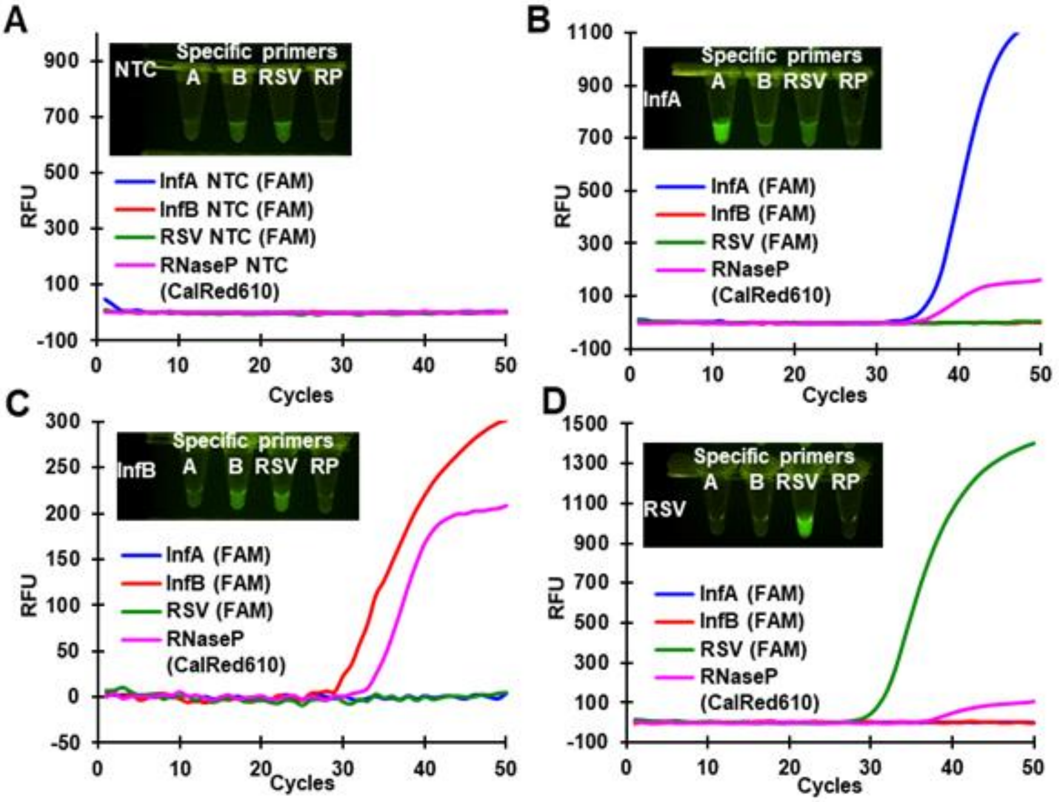

Figure S2. Direct detection of influenza A, B and RSV using unprocessed samples in VTM. Influenza A, B and RSV clinical specimens were diluted 100 times in VTM to mimic low viral load samples. Each samples were stored separately in a 2-mL aerosol generating vial. The content of each vial was sprayed into a row of four-well PCR tubes each containing InfA, InfB, RSV or RNaseP (RP) primers/probes. InfA, InfB, and RSV hydrolysis probes were tagged with FAM dye, and the RP probe was tagged with CalfluorRed 610 (CalRed610) dye. A - NTC, B - InfA clinical specimen sprayed, C - InfB clinical specimen sprayed, D - RSV clinical specimen sprayed. Real-time PCR was able to correctly detect the presence of the respective viral RNA and the human DNA (RP being positive in all three reactions except NTC). Inserts in each real-time PCR plot – Images of PCR tubes taken under blue LED illumination with an amber-color filter to capture the FAM dye's fluorescence. We note that the RSV probe gave higher background than InfA and InfB but the florescence signal in the tubes did not change from cycle 0 to cycle 50 so there's no non-specific amplification. .

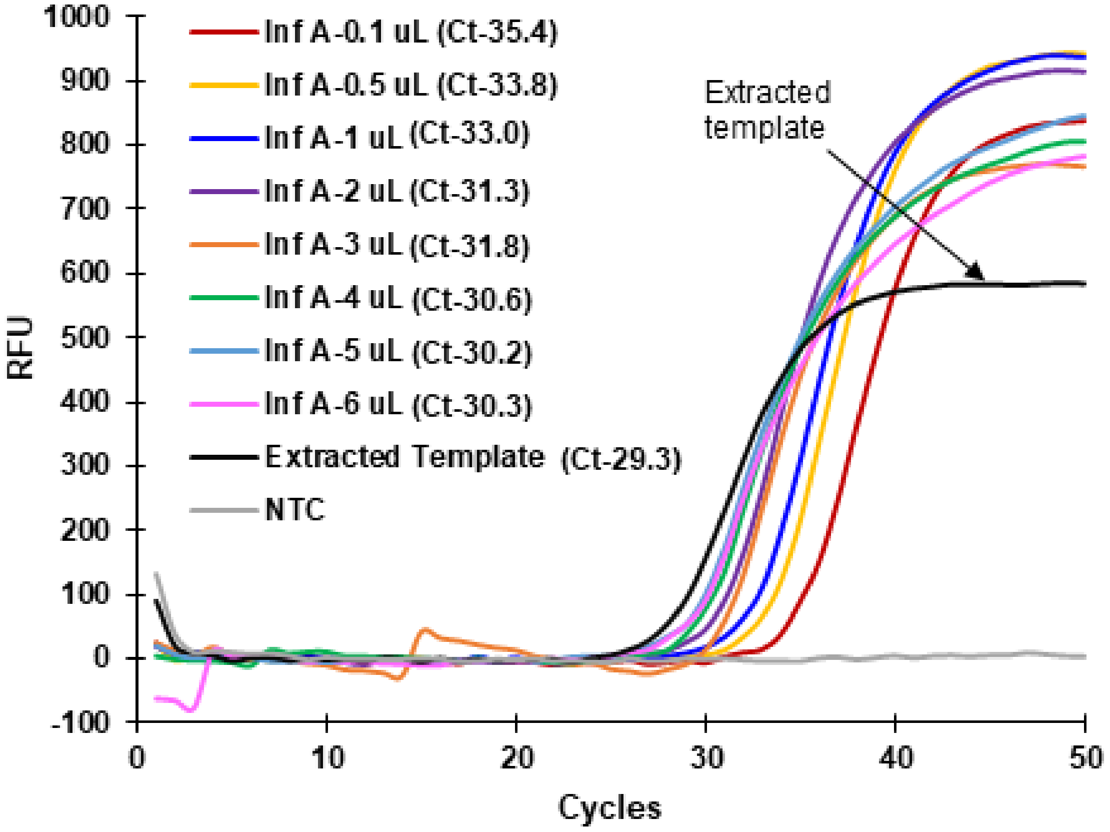

Figure S3. Using clinical samples without RNA extraction steps did not inhibit the reaction. In a 20-μL RT-qPCR run, 0.1, 0.5, 1, 2, 3, 4, 5 and 6 μL of InfA positive clinical specimens were directly added. Purified nucleic acid template (4 μL) isolated from a Promega Maxwell device was also amplified. The Ct difference between the purified template (Ct at 29.3) and the directly spiked clinical samples (i.e., 4 μL, 5 μL and 6 μL with Cts of 30.6, 30.2 and 30.3, respectively) is minimal. .

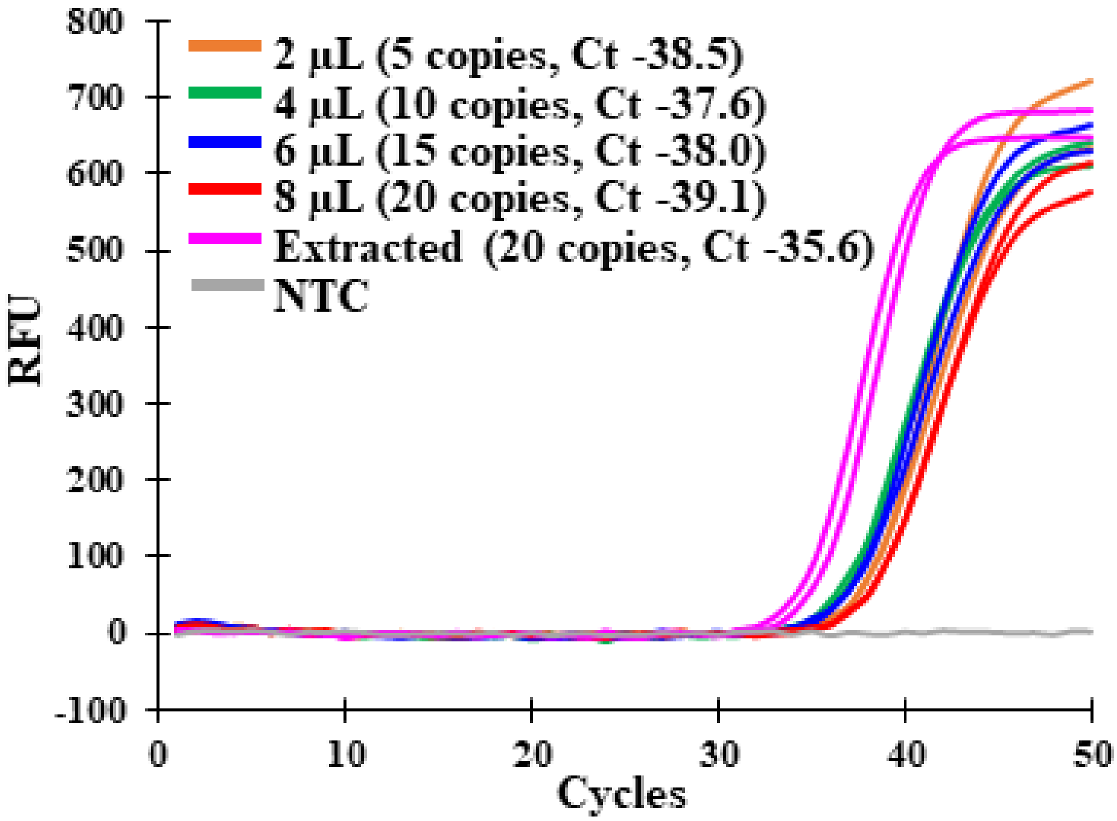

Figure S4. Low concentrations of SARS-CoV-2 reference material in VTM are detected without an RNA extraction step. SARS-CoV-2 non-replicative, inactivated viral particles (AccuPlex SARS-CoV-2 reference material from SeraCare containing 5,000 viral particles/mL) were diluted and mixed in an equal amount of VTM to mimic clinical samples (2500 viral particles/mL). 2 µL, 4 µL, 6 µL and 8 µL of this sample were directly spiked into a 20 µL PCR reaction mix containing SARS-CoV-2, N1 targeting primer and probe set and amplified alongside templates extracted in Promega Maxwell device (100 µL input and 100 µL eluate). RT-qPCR can detect all the directly spiked samples without any sample preparation samples (n = 2). The Ct difference between the purified template (Ct at 35.6) and the directly spiked clinical samples is rather small (e.g., 2 µL, 4 µL and 6 µL of clinical samples with Cts of 38.5, 37.6, and 38.0, respectively shown). Even when 8 µL of unprocessed sample was used in a 20-µL reaction, the sample was determined as positive (Ct = 39.1). This shows that unprocessed samples with low concentration of viral RNA (Ct values > 35) can still be correctly identified by RT-PCR.

Table S1. RT-qPCR detection of SARS-CoV-2 plasmid in VTM.

Target	SARS-CoV-2 Plasmid (Average Ct)	SARS-CoV-2 Plasmid in VTM (Average Ct)
N1	33.6	33.8
N2	34.4	34.6
**N3**	34.3	34.9

Table S1. RT-qPCR detection of SARS-CoV-2 plasmid in VTM. SARS-CoV-2 plasmid in VTM did not show any PCR inhibition when compared to plasmid alone. The CDC positive control plasmid for SARS-CoV-2 (from IDT) was diluted in VTM or TE buffer (control) to get 100,000 copies per mL (100 copies/μL). 4 μL of SARS-CoV-2 plasmids in VTM and in TE buffer were added to a 20 μL PCR reaction mix targeting N1, N2 and N3 genes for the detection of SARS-CoV-2. The Ct values of both the control and plasmid in VTM are very similar, indicating that the sample preparation step can be omitted in samples (from symptomatic patients) that produce high Ct values.
